# Cylindrical orifice testing in laminar flow with the orifice diameter ratio *β* = 0.5

**DOI:** 10.1038/s41598-023-42451-0

**Published:** 2023-09-18

**Authors:** Anna Golijanek-Jędrzejczyk, Andrzej Mrowiec

**Affiliations:** 1 Faculty of Electrical and Control Engineering, Gdańsk University of Technology, 80-233 Gdansk, Poland; 2 Polytechnic Faculty, Calisia University, 62-800 Kalisz, Poland

**Keywords:** Engineering, Energy science and technology

## Abstract

The paper presents the results of an experimental study of a cylindrical orifice with the orifice diameter ratio *β* = 0.5 and the flow opening length-to-diameter ratio *L*/*d* = 1, with hydraulic oil flowing in the DN50 measuring channel. The measurements of the values characterising the oil flow were made in the laminar flow regime, for the Reynolds numbers ranging between *Re* = 100 to 950. Based on the experimental tests, standard flow characteristics were created for four kinematic viscosity values in the range of 13.4–33.3 cSt, for which the average value of the discharge coefficient *C* in the tested flow rate range of *q*_*v*_ < 0.5 dm^3^/s was determined.

## Introduction

The volumetric flow rate is the amount of fluid flowing through the cross-section of a hydraulic flow channel per unit of time. The parameters affecting the physical properties of the fluid (including viscosity and density) are pressure and temperature. They are measured directly in the flow channel, which allows for determining the viscosity and density of the flowing fluid^1^.

Based on both the authors' experience from simulation and experimental studies and the available literature on the subject^2–4^, the centric orifice method is the most popular and versatile method for measuring fluid flow, which has been described and standardised in detail^5^. It is estimated to be the most widely used method in industrial measurement (it has captured more than 40% of the market^2^) in industries such as oil and gas, chemical, nuclear and power generation. For example, in China's oil and gas industry, flowmeters of this type account for about 95% of all flowmeters used in industrial installations^2^.

In this method, the flow rate is proportional to the root of the measured static pressure difference upstream and downstream of the orifice.

The standard orifice centric flowmeter is cheap to manufacture, inexpensive to operate and reliable for measuring the flow rate of flowing fluid in industrial applications^6,7^.

It is estimated that among all fluid flow measurements, orifice measurements still account for about 50%. Standard ISA orifices with sharp edges at the inlet have already been tested and standardised. For large Reynolds numbers (*Re* > 10,000) and the pipeline diameter *D* ≥ 50 mm, the value of the discharge coefficient *C* is practically constant and is usually presented in the form of tables (standard) or from the empirical Reader–Harris/Gallagher formula recommended by the standard^5^.

The discharge coefficient *C* is a parameter characterising the ratio of the actual value of the flow rate of a liquid flowing through a measuring orifice to the corresponding value calculated from a theoretical flow model. The numerical value of *C* is related to the shape of the orifice (its type) but also depends on the parameters of the orifice, the fluid flow conditions, the measuring instruments and the hydraulic installation.

In the range of Reynolds numbers smaller than 10,000, standard sharp-edged centric orifices are characterised by a significant variation of the discharge coefficient *C* as a function of Reynolds number, thus posing significant metrological problems and cannot be used in this case. In Ref.^8,9^, the authors attempted to determine the discharge coefficient *C* for low Reynolds number values, showing its non-linearity depending on the orifice diameter ratio *β* in this range of numbers *Re*.

This has motivated the researchers to develop new non-standardised orifices which, although requiring individual calibration, will be characterised by a constant discharge coefficient *C* in the viscous fluid flow range^10–12^. The oil viscosity, which changes with temperature, leads to relatively low Reynolds numbers characteristic of laminar and developing turbulent flows^13,14^.

An orifice with a conical inlet, for which the discharge coefficient remains constant in low Reynolds numbers (*R*e > 80), is handy for industrial flow rate measurements of viscous fluids^15,16^.

Another orifice allowing flow measurements at low Reynolds numbers (*Re* > 250) is the quadrant orifice, in which the radius of the rounding is related to the orifice diameter ratio *β*^17^.

Based on the symmetrical orifice with sharp-edges encountered in metrology (the so-called boundary orifice), which allows flow measurement in both directions, it was decided to use its thickened version as a cylindrical orifice for the study.

Despite its use in measurement systems, there are still a small number of publications in the scientific literature^18^ on cylindrical nozzles (elongated sharp-edged cylindrical orifices). The results presented therein mainly relate to turbulent flows, where only gas or water is used as the medium. That is why another orifice solution has been developed that allows the measurement of fluid flow rate at low Reynolds numbers, in the area of laminar and developing turbulent flows.

One example application is in the pharmaceutical industry, where the effect of the size of the flow of diluents through cylindrical orifices (in a tabletting matrix) as a function of their length and diameter was investigated using modelling dependent on the adopted calculation algorithm^19^.

In the literature^20^, the results of numerical tests can be found for low Reynolds numbers in a cylindrical orifice, in which the flow opening length-to-diameter ratio is *L*/*d* ≤ 15. Further experimental results have confirmed the results of the simulation tests.

However, for fluid flow measurement, the first cylindrical orifices were developed in 1938 for a pipeline with an internal diameter of *D* = 40 mm and an orifice diameter ratio *β* of 0.1–0.7^21^. Experimental flow tests were carried out for them, showing the distribution of the discharge coefficient characteristics as a function of the Reynolds number 270 > *Re* > 550,000.

On the other hand, publication^22^ presents the range of applicability of the cylindrical orifice and flow tests^23^ with a variation of the orifice diameter ratio *β* = 0.3–0.8 in pipelines with internal diameters *D* equal to 15, 25, 32 and 40 mm for Reynolds numbers *Re* > 1500.

Thus, in paper^18^, the author, based on his own research and that of other researchers^19,21^, presented empirical formulae for calculating the discharge coefficient *C* at different values of Reynolds number (*Re* > 500), depending on the adopted value of the orifice diameter ratio *β* in the range 0.1–0.7.

The paper^24^ estimated discharge coefficient magnitudes from numerical tests for air and water flowing through thin and thick (cylindrical) orifices with sharp edges.

Meanwhile, in the paper^25^, the tests performed for cylindrical orifices with small flow orifice diameters and the *L*/*d* ratio in the range of 1–50 have shown that the discharge coefficient *C* is correlated with the Reynolds number values.

The authors in^26^ compared the results of the discharge coefficient obtained from experimental tests and CFD numerical simulations for cylindrical damping holes in hydraulic oil dampers. They presented an analysis allowing the theoretical calculation of this coefficient to be improved and refined by about 17% in flow-damping systems.

In Ref.^27^, based on numerical and experimental studies of orifices with the ratio *L*/*d* = 2, it was demonstrated that additional corrections taking into account the occurrence of cavitation should be introduced to the theoretically determined discharge coefficient *C* in a high-pressure flow to achieve greater accuracy of this parameter.

However, it is more common in the literature to find publications analysing the issue of cavitation supported by experimental studies with the fluid flowing in the cylindrical orifice area for low or high-pressure values^28,29^.

Another example of this type of research can be found in experimental studies^30^, which evaluated the effect of the inlet geometry of elongated orifices (sharp-edged and chamfered) of rocket engine injectors in fluid flow. The paper^30^ assessed the impact of orifice diameter and thickness on the formation of cavitation phenomena in turbulent flow, improving liquid fuel atomisation in the combustion chamber.

To summarise, several older articles (more than 40 years old) deal with studies similar to those carried out by the authors. However, it should be mentioned that, first of all, there are only a few of them, and, secondly, there are still no results concerning flow tests carried out in an oil installation with a pipe diameter of ø 50. The published research^21,23^ was performed for installations with a water medium and only up to a maximum pipeline diameter of ø 40.

The experimental investigations described in this paper aimed to determine the discharge coefficient for a selected cylindrical orifice with the orifice diameter ratio *β* = 0.5 and the flow opening length-to-diameter ratio *L*/*d* = 1 in pipeline DN50. The measurements were carried out in the laminar range of the hydraulic oil flow (100 < *Re* < 1000) for different viscosity values determining the flow characteristics. As the available literature lacks metrological analyses and experimental tests for flows of fluids with a viscosity higher than that of water, the presented test results will supplement this knowledge, thus confirming the novelty of the conducted tests.

## Research object

When designing the orifice for testing, it was assumed that the oil Hydrol L-HL 22 with an average density of *ρ* = 864 kg/m^3^ flows through the pipeline with an inner diameter of ø 50 mm. This oil will flow with the maximum flow rate *q*_v_ ≤ 0.5 dm^3^/s, and the pressure drop *Δp* at the orifice will not exceed 1.2 kPa.

Based on the above defined criteria, a cylindrical orifice has been designed which, according to the literature, can be used to measure viscous fluid flows at low Reynolds numbers^18^. For the designed orifice with the flow section length of 25 mm, the orifice diameter ratio *β* was assumed to be equal to 0.5.

**Figure 1:**
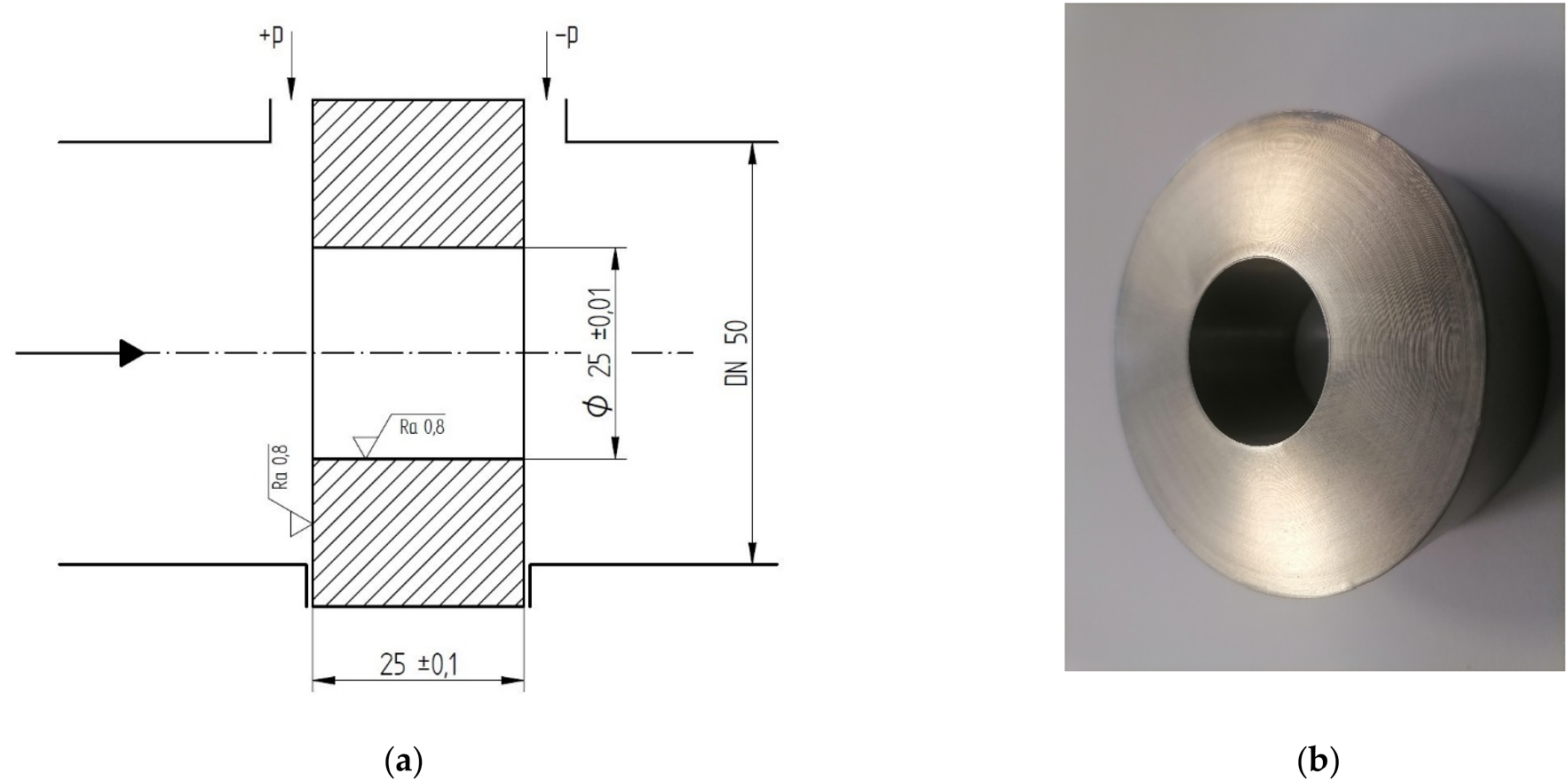
The cylindrical orifice with orifice diameter ratio *β* = 0.5: (**a**) geometrical dimensions, (**b**) view.

Figure 1a shows the longitudinal cross-section of the cylindrical orifice with geometrical dimensions allowing its fabrication and the assumed roughness values on its critical surfaces.

A prototype of the above orifice was made of stainless steel, as shown in Fig. 1b.

## Experimental stand

The flow rate measurements intended to determine *q*_ve_ = f(*Δp*) characteristics for different values of oil kinematic viscosity were conducted on a modified test stand (HYDAC hydraulic power pack).

The oil flow rate in the hydraulic system was changed smoothly using an inverter controlling the speed of the electric motor within the range of 435–2750 rpm. The motor (M) was connected via a claw coupling to an internal gear pump with a maximum capacity of *q*_v_ = 0.5 dm^3^/s at nominal speed.

**Figure 2:**
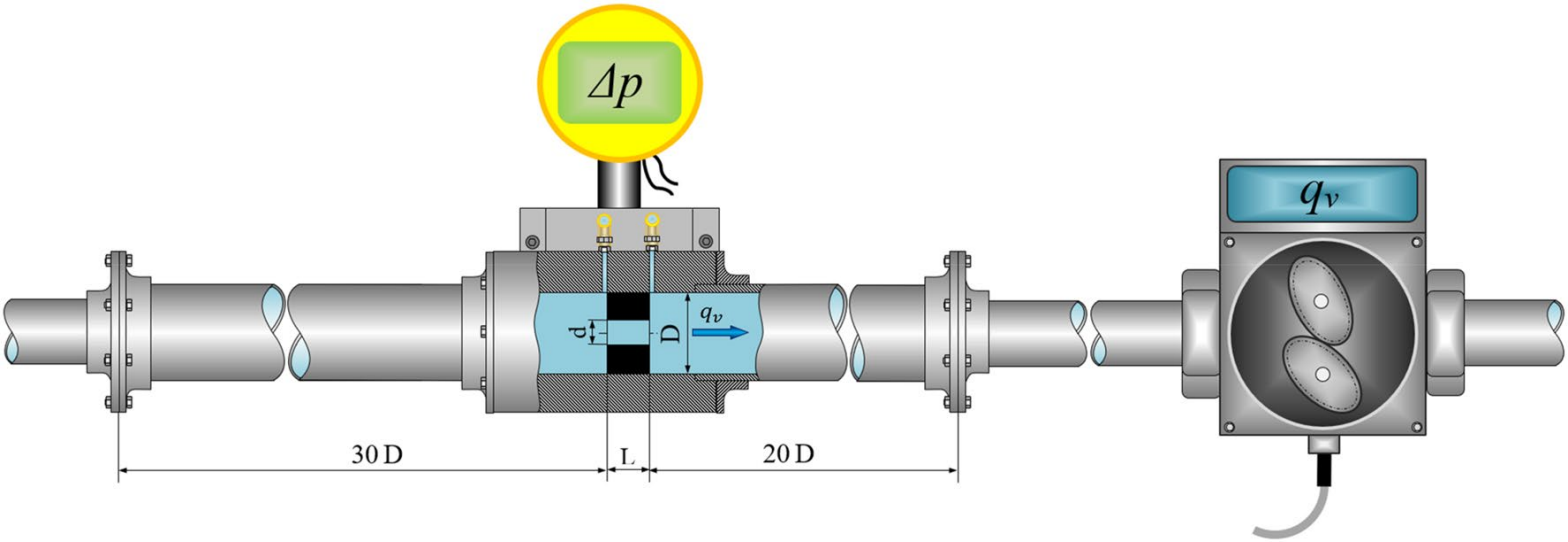
Schematic of the hydraulic measuring system.

Figure 2 shows the schematic of the hydraulic measuring system. As can be seen, the oil first flows through the measuring system with the cylindrical orifice under test and then enters the oval flowmeter NC4.

In the hydraulic system shown, the NC4 flowmeter was adopted as the standard for measuring the flow rate of the flowing oil. Changes in the kinematic viscosity of the oil were controlled by changing its temperature.

A massive steel oil tank with a capacity of approximately 250 dm^3^ was the base of the entire hydraulic system of the test stand. A UTU-2 water ultra-thermostat located on the test bench was used to stabilise the set temperature of the flowing oil. It was connected to a tubular heat exchanger, made in the form of a copper heating coil immersed in the oil filling the tank.

The oil temperature in the tank was measured continuously using a Pt100 resistance temperature sensor in a steel case with a terminal head connected to an AT-2-type temperature transmitter. The temperature transmitter was programmed for a measuring range of 0–80 °C, with a standard output signal of 4–20 mA DC.

As the reference standard for measuring the oil flow rate, an oval wheel flowmeter type NC4 was used. This flowmeter has the limiting error of the measured value equal to *Δq*_v_ ≤ 0.25% in the measuring range of *q*_v_ = 0.003–0.83 dm^3^/s. The differential pressure *Δp* arising at the orifice was measured in a point-differential manner using a differential pressure transmitter type APR-2000/ALW. Its limiting error *Δp* ≤ 0.15% was programmed for the differential pressure measurement range *Δp* = 0–1.2 kPa, with the standard output current signal of 4–20 mA.

## Results of measurements

The following parameters were recorded during the experimental tests: the volumetric rate qv of the hydraulic oil flow through the orifice, the pressure difference *Δp* across the orifice, and the fluid temperature *T*.

Each averaged result of a given parameter was calculated from 300 individual results of this parameter, measured with the sampling step of 3 s.

Based on the equation:

1 $$C = \frac{{4 \cdot q_{v} \cdot \sqrt {1 - \beta^{4} } }}{{\pi \cdot \varepsilon \cdot d^{2} \cdot \sqrt {\frac{2 \cdot \Delta p}{\rho }} }}$$

the discharge coefficient *C* of the tested cylindrical orifice was calculated for each sample, taking into account the average density of the flowing oil at the test temperature.

The results of the performed experimental measurements and calculations are given in Table 1.

**Table 1:** Experimental results.

*T*_*śr*_ = 48.89 [°C]				*T*_*śr*_ = 44.35 [°C]			
*ν*_*śr*_ = 13.37 [mm^2^/s = cSt]				*ν*_*śr*_ = 15.27 [mm^2^/s = cSt]			
*ρ*_*śr*_ = 846.2 [kg/m^3^]				*ρ*_*śr*_ = 849.3 [kg/m^3^]			
*q*_*v*_	*Δp*	*Re*	*C*	*q*_*v*_	*Δp*	*Re*	*C*
[dm^3^/s]	[kPa]	[—]	[—]	[dm^3^/s]	[kPa]	[—]	[—]
0.1695	0.0824	323.2	0.7576	0.1698	0.0849	283.5	0.7496
0.2208	0.1327	419.2	0.7783	0.2206	0.1362	366.2	0.7688
0.2720	0.1944	518.0	0.7918	0.2725	0.1995	453.1	0.7846
0.3060	0.2426	583.0	0.7973	0.3054	0.2458	510.0	0.7922
0.3397	0.2961	647.3	0.8014	0.3390	0.2992	566.4	0.7970
0.3722	0.3527	709.8	0.8045	0.3734	0.3595	623.1	0.8008
0.4063	0.4182	775.0	0.8066	0.4076	0.4253	681.1	0.8037
0.4415	0.4923	840.6	0.8078	0.4408	0.4945	737.0	0.8061
0.4833	0.5896	922.6	0.8080	0.4832	0.5916	806.9	0.8080

*T*_*śr*_ = 35.10 [°C]				*T*_*śr*_ = 25.11 [°C]			
*ν*_*śr*_ = 21.00 [mm^2^/s = cSt]				*ν*_*śr*_ = 33.25 [mm^2^/s = cSt]			
*ρ*_*śr*_ = 855.6 [kg/m^3^]				*ρ*_*śr*_ = 862.5 [kg/m^3^]			
*q*_*v*_	*Δp*	*Re*	*C*	*q*_*v*_	*Δp*	*Re*	*C*
[dm^3^/s]	[kPa]	[—]	[—]	[dm^3^/s]	[kPa]	[—]	[—]
0.1715	0.0932	208.5	0.7251	0.1706	0.1043	121.5	0.6851
0.2212	0.1458	268.3	0.7478	0.2221	0.1611	173.6	0.7207
0.2715	0.2101	328.6	0.7645	0.2724	0.2320	200.5	0.7332
0.3064	0.2613	371.4	0.7736	0.3061	0.2852	229.1	0.7428
0.3403	0.3175	412.2	0.7795	0.3399	0.3424	260.2	0,7529
0.3731	0.3759	451.8	0.7854	0.3735	0.4043	294.5	0.7612
0.4082	0.4431	494.8	0,7916	0.4079	0.4722	329.2	0.7689
0.4426	0.5159	537.6	0.7954	0.4420	0.5467	366.0	0.7742
0.4835	0.6090	588.1	0.7997	0.4836	0.6415	412.4	0.7819

**Figure 3:**
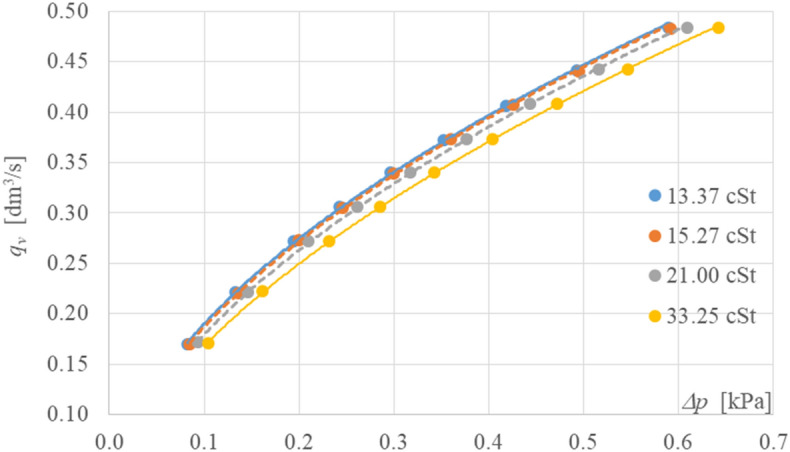
Flow characteristics *q*_*ve*_ = f*(Δp)* as a function of kinematic viscosity.

The above results made the basis for creating flow characteristics *q*_ve_ = f(*Δp*) for the cylindrical orifice tested. The obtained characteristics are shown in Fig. 3, along with the base points used for their creation.

The two extreme curves of the flow characteristics for kinematic viscosities of 13.37 mm^2^/s and 33.25 mm^2^/s within the flow rate range below *q*_v_ < 0.5 dm^3^/s are marked as continuous trend lines, while the two other point trend lines situated between them characterize the intermediate kinematic viscosities for which the measurements were also made, as shown in Table 2.

**Table 2:** Parameters of potentiated trend lines.

Kinematic viscosity	*α*	*γ*	*R*^*2*^
13.37 cSt	0.6462	0.5323	0.9996
15.27 cSt	0.6462	0.5388	0.9997
21.00 cSt	0.6392	0.5516	0.9999
33.25 cSt	0.6257	0.5711	0.9999

The potentiated trend lines shown in Fig. 3 can be described algebraically by the general relation:

2 $${q}_{ve}=\alpha \cdot \Delta {p}^{\gamma }$$

Table 2 summaries the trendline parameters as a function of the averaged values of kinematic viscosity of the flowing oil represented by measurement points (*q*_v_, *Δp*), together with the corresponding values of the determination coefficient *R*^2^.

It can be seen from the trendline parameter values shown in Table 2 that the parameters are strongly dependent on the viscosity of the hydraulic oil flowing in the measurement system.

Equation (2) shows that as the kinematic viscosity value increases, the value of the proportionality coefficient α decreases and the value of the power exponent *γ* increases. The value of the power exponent differs significantly from the theoretical value of *γ* = 0.5. It was, therefore, decided to fit another function with a power exponent *γ* = 0.5 (theoretical value)^5^ with the smallest possible error to the flow characteristic determined by the trend line with a kinematic viscosity of 13.37 cSt, (the power exponent is *γ* = 0.5323).

These flow values for this function were calculated using the iterative method by adapting a theoretical power function with exponent *γ* = 0.5. For this new function, the proportionality coefficient has the value *α* = 0.626. Between the experimental curve (*α* = 0.6462; *γ* = 0.5323) and the determined new theoretical power function (*α* = 0.626; *γ* = 0.5), the fit error does not exceed 0.9% at the measured backpressure at the orifice in the range of *Δp* = 0.1–0.6 kPa.

Based on the power function determined in this way, the theoretical values of the mass flow rate *q*_*vo*_ as a function of *Δp* were calculated, for which the theoretical value of the discharge coefficient was determined from Eq. (1), where *C* is 0.8036.

**Figure 4:**
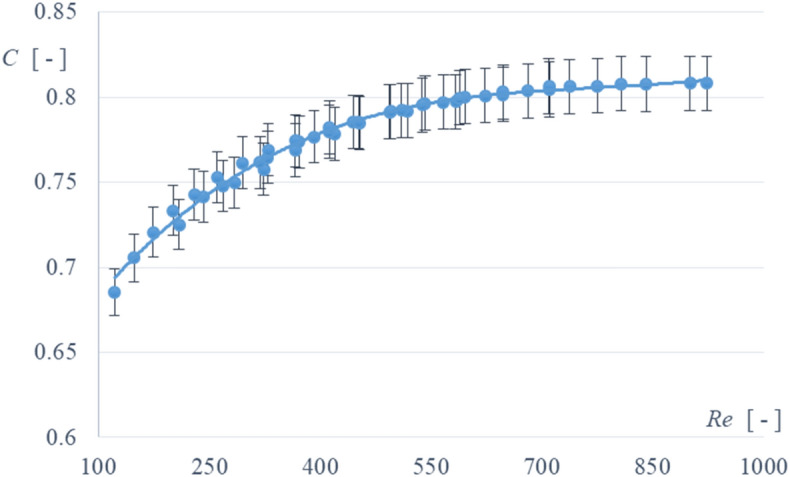
Distribution of discharge coefficient vs Reynolds number.

Using the results obtained in experimental tests with flowing oil for four different kinematic viscosities (Table 1), the discharge coefficient *C* values will be calculated. Its distribution as a function of Reynolds numbers is shown in Fig. 4. For each point creating the graph *C* = f(*Re*) in Fig. 4, a tolerance area expressed by an error bar of 2% was added. This value was estimated based on the literature on standardised and non-standardised holes^31^: with a conical and quadrant inlet and based on our own research on determining the uncertainty of the flow coefficient for a multi-hole^32^.

It can be observed in the plot shown in Fig. 4 that starting from *Re* = 550, the curved fit line to the discharge coefficient values calculated from the experiment begins to straighten, which makes it more flat.

## Discussion

From the literature analysis, a few publications present experimental studies to determine the discharge coefficient *C* for measured elongated cylindrical orifices. In the published articles, experimental tests were carried out in pipelines with a maximum diameter not exceeding DN40^18,21,23^ with a flowing water stream.

The verification of these orifices in water flow systems is made possible by tests from low Reynolds numbers (*Re* approx. 1400), at which very low pressure drop values across the orifice occur, resulting in increased measurement errors.

Therefore, to increase the accuracy of the measurements made at lower Reynolds number values, the authors carried out experimental tests in a DN50 measuring channel with hydraulic oil flowing through, the viscosity of which is several times that of water. This allowed a significant reduction in Reynolds number values at the same orifice pressure accumulations (or flow rate values) as for water.

In a DN50 channel, an elongated cylindrical flow orifice with an orifice diameter ratio *β* = 0.5 and a length (thickness) ratio to the flow orifice diameter *L*/*d* = 1 was tested. The *Δp* and *q*_*v*_ results obtained from the experiment were averaged over the measurement series. Based on these, the discharge coefficient *C* calculations were made at low Reynolds number values (*Re* = 100–950), shown in Fig. 4 as dots with the bars of the adopted tolerance field of 2%.

The adopted tolerance field in the range of Reynolds numbers *Re* = 450–950 contains the value of the discharge coefficient *C* = 0.8036, which was calculated from relation (1) using the theoretical (engineering) curve described by a power function (2) for which the exponent of the power *γ* = 0.5 and the proportionality factor *α* = 0.626.

However, this calculated theoretical value of the discharge coefficient is 0.55% lower than the value reported in the literature (*C* = 0.808) for an elongated cylindrical orifice with an orifice diameter ratio *β* = 0.5, but with a larger ratio *L*/*d* = 2.12. The above value was determined in a DN 40 pipeline for flowing water at Reynolds number *Re* ≥ 1400^18,23^. For the cylindrical orifice tested, a similar value for the discharge coefficient *C* was obtained for a quadrant orifice with an orifice diameter ratio *β* = 0.5 (*C* = 0.802)^31^.

**Figure 5:**
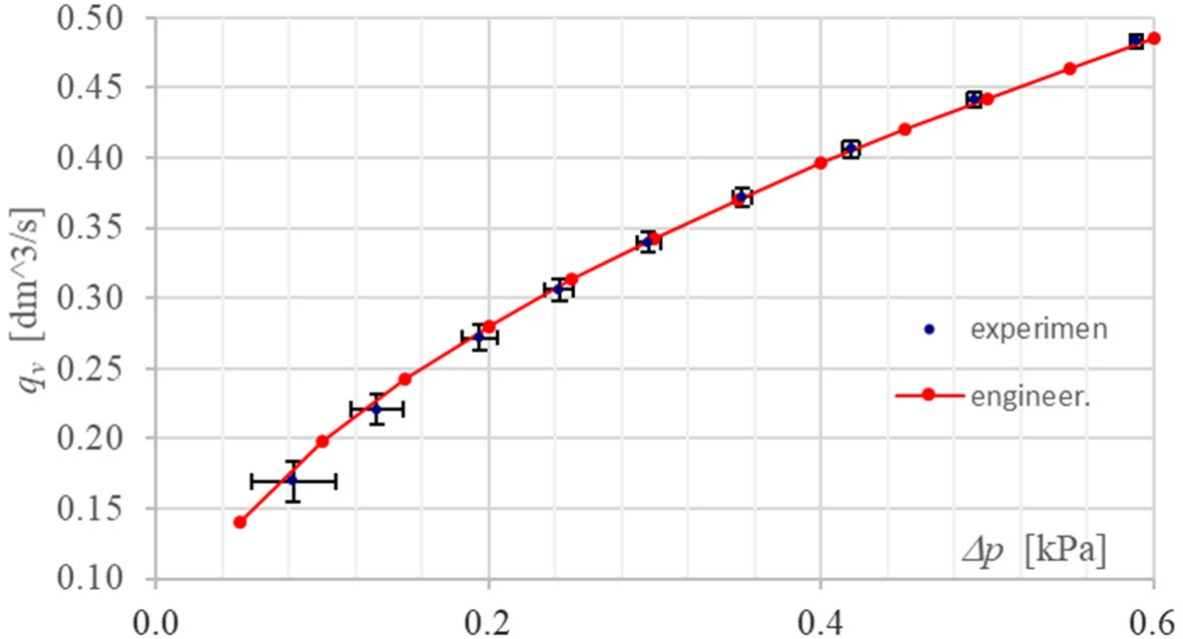
Comparison of flow characteristics for a cylindrical orifice: experimental and engineering.

Figure 5 shows the flow characteristics as averaged experimental points for flowing hydraulic oil with a kinematic viscosity of 13.37 cSt.

The averaged measurement points of flow rate *q*_*v*_ and differential pressure *Δp* through the test hole are plotted against the expanded uncertainty bars of both these values (estimated from the type B uncertainty due to imperfections in the measuring instruments used to measure these quantities).

The (engineering) flow characteristic was calculated for the theoretically determined discharge coefficient *C* = 0.8036 and is shown as points connected by a solid line. It can be seen from the graph (Fig. 5) that this characteristic is within the tolerance range limited by the expanded uncertainty bars of the two values (determined from the Type B expanded uncertainty of the measurement apparatus used).

## Conclusions

The paper presents the results of experimental tests for hydraulic oil flowing in a DN50 flow channel through a cylindrical orifice with the diameter ratio of *β* = 0.5 and the flow opening length (thickness)-to-diameter ratio *L*/*d* = 1.

It can be concluded from the presented results of the tests performed on a cylindrical orifice (Fig. 1) for corner tappings that in the tested range of Reynolds numbers (*Re* = 450–950), a constant value of the discharge coefficient *C* equal to 0.8036 can be assumed, with a theoretical exponent *γ* = 0.5 and a kinematic viscosity in the range of 13–15 mm^2^/s (cSt). In this range of *Re* numbers, this value is within the accepted 2% tolerance for the *C* = f(*Re*) characteristic determined from experimental tests (Table 1).

In industrial practice, (oil) hydraulic systems operate at 40–50 °C. Under such oil flow conditions, an orifice flow meter with a cylindrical orifice with the orifice diameter ratio* β* = 0.5 can be used to monitor system operation—as a cheap and reliable device.

The experimental studies allowed the Reynolds number limit *Re*_min_ to be reduced from a value of 1400^18,23^ to a value of 450 (with an assumed tolerance of 2%), from which a cylindrical orifice with the orifice diameter ratio *β* = 0.5 can already be used to measure the flow rate.

Further experimental studies are planned for laminar flow (*Re* > 1000), including the developing turbulent flow.

## Data Availability

The datasets used during the current study are available from the corresponding author on reasonable request.

## Abbreviations

*D*: Pipe diameter [m]
*d*: Orifice diameter [m]
*L*: Length of the flow hole in the orifice [m]
*T*: Fluid (oil) temperature [°C]
*a*: Slope [–]
*Re*: Reynolds number [–]
*b*: Intercept [dm^3^/s]
*q*_*v*_: Volumetric flow [dm^3^/s]
*C*: Discharge coefficient [–]
*ε*: Expansion coefficient, for oil *ε* = 1
*Δp*: Difference pressure [Pa]
*α*: Proportionality coefficient [m^4^/(kg·m)^0.5^]
*β*: Orifice diameter ratio [–]
*γ*: Power coefficient [–]
*ρ*: Fluid density [kg/m^3^]
*ν*: Kinematic viscosity [cSt = mm^2^/s]
*u(a)*: Standard uncertainty of determination of parameter *a*
*u(b)*: Standard uncertainty of determination of parameter *b*

## References

[1] Liptak BG (2003). Process measurement and analysis.

[2] Dong J, Jing C, Peng Y, Liu Y, Ren H, Liu X (2018). Study on the measurement accuracy of an improved cemented carbide orifice flowmeter in natural gas pipeline. Flow Meas. Instrum..

[3] Abd HM, Alomar OR, Mohamed IA (2019). Effects of varying orifice diameter and Reynolds number on discharge coefficient and wall pressure. Flow Meas. Instrum..

[4] Sravani V, Santhosh Krishnan V (2022). Parametric analysis of orifice plates on measurement of flow: A review. Ain Shams Eng. J..

[5] ISO 5167-2:2003: Measurement of fluid flow by means of pressure differential devices inserted in circular cross-section conduits running full-Part 2: Orifice plates. (2003).

[6] Martines, N. Manual de medição de vazão: através de placas de orifício, bocais e venturis. Interciência. (1998).

[7] Panton, R. L. *Incompressible flow*. John Wiley & Sons, Inc. 10.1002/9781118713075 (2013).

[8] Hollingshead CL, Johnson MC, Barfuss SL, Spall RE (2011). Discharge coefficient performance of Venturi, standard concentric orifice plate, V-cone and wedge flow meters at low Reynolds numbers. J. Pet. Sci. Eng..

[9] Miller, R. W. Flow measurement engineering handbook. ISSN 0142727X, 10.1016/0142-727x(83)90065-6 (1983).

[10] Kuratow, T. Venturi measurement of fuel oil flow rate at small Reynolds number values. Ph.D. Thesis, Silesian University of Technology, Poland, Gliwice. (1973).

[11] Ho YS, Leong TP (1985). Performance of conical entrance orifice plates at low Reynolds numbers. Int. J. Heat Fluid Flow.

[12] Bruner, R.F. An experimental and theoretical investigation of conic entrance orifice performance in the low Reynolds number domain. *DTIC Archive* (1972).

[13] Pozrikidis, C. Flow at low reynolds numbers. *Springer Science+Business Media New York*. 10.1007/978-1-4757-3323-5_9 (2001).

[14] Hasan D (2021). Effect of conical angle on the hydraulic properties of orifice plate flows: A numerical approach. Flow Meas. Instrum..

[15] Ho YS, Abdullah F (1995). Modeling the conical entrance orifice plate flow sensor. Trans. Inst. Meas. Control..

[16] PN/M-42377. Measurements of the fluid stream using measuring venturis. Guidelines for the selection of nozzles and orifices not covered by ISO 5167-1.

[17] British Standards BS 1042 (Part 1: Pressure Differential Devices): Measurement of Fluid Flow in Closed Conduits.

[18] Kremlewsk, P. P. Raschodimiery i scetciki kolicestva vescestv. Izd. Politechnika SaintPetersburg, (2002).

[19] Kachrimanis K, Petrides M, Malamataris S (2005). Flow rate of some pharmaceutical diluents through die-orifices relevant to mini-tableting. Int. J. Pharm..

[20] Jankowski TA, Schmierer EN, Prenger FC, Ashworth SP (2008). A series pressure drop representation for flow through orifice tubes. J. Fluids Eng..

[21] Koennecke W (1938). Neue Düsenformen für kleinere und mittlere Reynoldszahlen. Forschung auf dem Gebiete des Ingenieurs Wesens..

[22] Kabza, Z. *Measurements of fluid streams (Guide)*. Oficyna Wydawnicza Politechniki Opolskiej. Studies and monographs 90, Opole (1996).

[23] Kabza Z (1977). Durchfluss- und Expansionszahlen einer neuen Zylinderdüses. Brennstoff-Waerme-Kraft.

[24] Panda, S.K., & Patra, A. *Determination of Coefficient of Contraction of Orifice with Variation of Geometrical Parameter*. Theoretical, Computational, and Experimental Solutions to Thermo-Fluid Systems. Lecture Notes in Mechanical Engineering 413–421 (Springer, Singapore). 10.1007/978-981-33-4165-4_38 (2021).

[25] Ramamurth K, Nandakumar K (1999). Characteristics of flow through small sharp-edged cylindrical orifices. Flow Meas. Instrum..

[26] Ding YW, Wei XH, Nie H, Li YP (2018). Discharge coefficient calculation method of landing gear shock absorber and its influence on drop dynamics. J. Vibroeng..

[27] Ebrahimi B, He G, Tang Y, Franchek M, Liu D, Pickett J, Springett F, Franklin D (2017). Characterization of high-pressure cavitating flow through a thick orifice plate in a pipe of constant cross section. Int. J. Therm. Sci..

[28] Stanley C, Barber T, Milton B, Rosengarten G (2011). Periodic cavitation shedding in a cylindrical orifice. Exp. Fluids.

[29] Espositoa C, Peveroni L, Gouriet JB, Steelant J, Vetrano MR (2021). On the influence of thermal phenomena during cavitation through an orifice. Int. J. Heat Mass Transfer..

[30] Kiaoulias DN, Travis TA, Moore JD, Risha GA (2019). Evaluation of orifice inlet geometries on single liquid injectors through cold-flow experiments. Exp. Thermal Fluid Sci..

[31] ISO/TR 15377:2007 Measurement of fluid flow by means of pressure-differential devices-Guidelines for the specification of orifice plates, nozzles and Venturi tubes beyond the scope of ISO 5167 (2007).

[32] Mrowiec A (2020). Uncertainty assessment for determining the discharge coefficient C for a multi-opening orifice. Appl. Sci..

